# Smoking and Suicide: A Meta-Analysis

**DOI:** 10.1371/journal.pone.0156348

**Published:** 2016-07-08

**Authors:** Jalal Poorolajal, Nahid Darvishi

**Affiliations:** 1 Modeling of Noncommunicable Diseases Research Center and Department of Epidemiology, School of Public Health, Hamadan University of Medical Sciences, Hamadan, Iran; 2 Psychological Counseling Center, Hamadan University of Medical Sciences, Hamadan, Iran; National Institute on Drug Abuse, UNITED STATES

## Abstract

**Background:**

Many studies have reported a positive association between smoking and suicide, but the results are inconsistent. This meta-analysis was carried out to estimate the association between smoking and suicidal ideation, suicide plan, suicide attempt, and suicide death.

**Methods:**

Major electronic databases including PubMed, Web of Science, Scopus, and ScienceDirect were searched until May 2015. The reference lists of included studies were screened too. Epidemiological studies addressing the association between smoking and suicidal behaviors were enrolled. The heterogeneity across studies was explored by Q-test and I^2^ statistic. The possibility of publication bias was assessed using Begg's and Egger's tests and Trim & Fill analysis. The results were reported based on risk ratio (RR) and odds ratio (OR) with 95% confidence intervals (CI) using a random-effects model.

**Results:**

We identified a total of 8062 references and included 63 studies with 8,063,634 participants. Compared to nonsmokers, the current smokers were at higher risk of suicidal ideation (OR = 2.05; 95% CI: 1.53, 2.58; 8 studies; I^2^ = 80.8%; P<0.001), suicide plan (OR = 2.36; 95% CI: 1.69, 3.02; 6 studies; I^2^ = 85.2%; P<0.001), suicide attempt (OR = 2.84; 95% CI: 1.49, 4.19; 5 studies; I^2^ = 89.6%; (P<0.001), and suicide death (RR = 1.83; 95% CI: 1.64, 2.02; 14 studies; I^2^ = 49.7%; P = 0.018).

**Conclusions:**

There is sufficient evidence that smoking is associated with an increased risk of suicidal behaviors. Therefore, smoking is a contributing factor for suicide. Although this association does not imply causation, however, smoking prevention and cessation should be the target of suicide prevention programs.

## Introduction

Each suicide is a tragedy. Every 40 seconds a person dies from suicide somewhere in the world. The estimated global burden of suicide is over 800,000 deaths per year [1]. Suicide accounted for 1.4% of total mortality and 15% of injury mortality of the world in 2012 [2]. These figures underestimate the problem. For each suicide related death, there are approximately 10 to 40 attempted suicides [3]. In addition, a lot of people with suicidal thoughts never seek services [4]. Suicides occur in all parts of the world and throughout the lifespan. It is the second leading cause of death in young people 15 to 29 years of age and is highest in persons aged 70 years or over for both men and women in most regions of the world [1]. Suicide is one of the greatest sources of premature death [5].

There is no single cause or stressor for suicide, but numerous psychological, social, biological, and cultural factors contribute to suicide [6–8]. Psychological disorders as well as alcohol and substance abuse disorders are among the major contributing factors for suicide [9–11]. Several epidemiological studies have reported an association between smoking and suicidal behaviors, but the results are inconsistent. A meta-analysis was conducted by Li et al [12] to estimate the overall association between smoking and suicide related death based on the studies published by May 2011. However, the association between smoking and other suicidal behaviors, such as suicidal ideation, suicide plan, and suicide attempt was not addressed. Furthermore, so far, several epidemiological studies have recently been conducted to address the relationship between smoking and suicidal behaviors. Therefore, an update and comprehensive meta-analysis is needed. We performed this meta-analysis based on current evidence to estimate the association between smoking and suicidal behaviors, including suicidal ideation, suicide plan, suicide attempt, and suicide death.

## Materials and Methods

### Protocol and registration

This review was approved and funded by the Vice-chancellor of Research and Technology, Hamadan University of Medical Sciences. This report was prepared according to the PRISMA, an evidence-based minimum set of items for reporting in systematic reviews and meta-analyses [13]. The supporting PRISMA checklist of this review is available as supporting information; see S1 PRISMA Checklist. The protocol was registered with the Prospero—Center for Reviews and Dissemination on 2 June 2015 (CRD42015022054), available from: http://www.crd.york.ac.uk/PROSPERO/display_record.asp?ID=CRD42015022054

### Eligibility criteria

The exposure of interest was smoking. Based on smoking habits, the participants were classified as non-smokers (never smoked or smoked less than 100 cigarettes), ex-smokers (smoked at least 100 cigarettes, but did not smoke in the past 30 days), or current smokers (smoked at least 100 cigarettes and smoked in the past 30 days [14]. We included the studies that reported cigarette habits, according to these categories or at least compatible with them. We excluded studies that did not distinguish between current smokers and former smokers (so called ever smokers), or assessed the association between suicide and age of initiation of smoking rather than a smoking habit itself, or compared the risk of suicide in high smokers versus low smokers, see S1 Excluded Studies With Reasons.

The outcome of interest was suicide. Suicidal behaviors were classified as suicidal ideation (seriously thought about committing suicide during past 12 months or life time), suicide plan (making a plan for committing suicide during the past 12 months or lifetime), suicide attempt (actually attempting suicide during the past 12 months or lifetime), and suicide death (dying of suicide) [15].

Observational studies, such as cohort, case-control, and cross-sectional studies, investigating the association between smoking and suicidal behaviors in general population were enrolled irrespective of language, date of publication, nationality, race, age, and gender. We excluded studies that did not discriminate among different types of suicidal behaviors or assessed the association between suicide and smoking in people with mental disorders.

### Information sources and search

Major electronic databases, including PubMed, Web of Science, Scopus, and ScienceDirect were searched until May 2015. The reference lists of the included studies were searched to identify additional studies.

The following search terms were used individually and in combination: (suicid* or *self-injurious behavior* or *self-mutilation* or *self-immolation* or *self-harm* or *self-inflicted* or *self-injury* or *self-slaughter* or *self-destruction*) and (*smoking* or *tobacco* or *cigarette* or *cigar*).

### Study selection

We combined search results from different databases using EndNote reference manager software and deleted duplicate records of the same report. Then, two authors screened independently titles and abstracts to remove ineligible studies. Disagreements were resolved by discussion. We retrieved the full text of the potentially eligible studies and examined full-text reports for further evaluation. In cases where there were multiple reports of the same study, we used the last published report.

### Data extraction

We extracted data from relevant studies using an electronic data collection form prepared in Stata software. We contacted study authors, where appropriate, to request further information, such as missing results. We extracted the following information: first author’s name, year of publication, country, language, population type (general population, conscripts/veterans), age of participants, gender, design of the studies (cohort, case-control, cross-sectional), suicidal behaviors (ideation, plan, attempt, completed), effect estimate (risk ratio, odds ratio), sample size, effect sizes and related 95% confidence intervals (CIs).

### Methodological quality

The methodological quality of the included studies was examined using Newcastle Ottawa Statement (NOS) Manual [16]. The NOS is a practical scale for assessing the quality of observational studies with their design and content. This scale includes a set of items and allocates a maximum of nine stars to the following domains: selection, comparability, exposure, and outcome. In this meta-analysis, the studies with six star-items or less were considered low-quality and those with seven star-items or more were considered high-quality.

### Heterogeneity and reporting biases

Heterogeneity was examined by chi-squared test [17] and its quantity was measured by the I^2^ statistic [18]. The possibility of publication bias was investigated by the Egger's [19] and Begg's [20] tests and Trim and Fill method [21].

### Summary measures

We used the risk ratio (RR) and the odds ratio (OR) with their 95% confidence intervals (CI) to express the association between smoking and suicidal behaviors. We analyzed data and reported the results based on a random-effects model [22]. We performed statistical analyses at a significance level of 0.05 using Stata software, version 11 (StataCorp, College Station, TX, USA).

### Subgroup analysis

We performed subgroup analysis according to the quality of included studies (high-quality and low-quality).

### Sensitivity analysis

In cases the between-study heterogeneity was high, we evaluated the source of heterogeneity using sequential algorithm [23]. According to this algorithm, one study was excluded from the calculations each time. The study that was responsible for the largest decrease in I^2^ was dropped. This process was repeated for a new set of n-1 studies. We continued by successively reanalyzing reduced sets of studies until I^2^ dropped below the intended threshold 50%. When there was a chance more than one omitted studies could result in I^2^ dropping below the desired threshold, we reported the minimum I^2^.

## Results

### Description of studies

**Fig 1:**
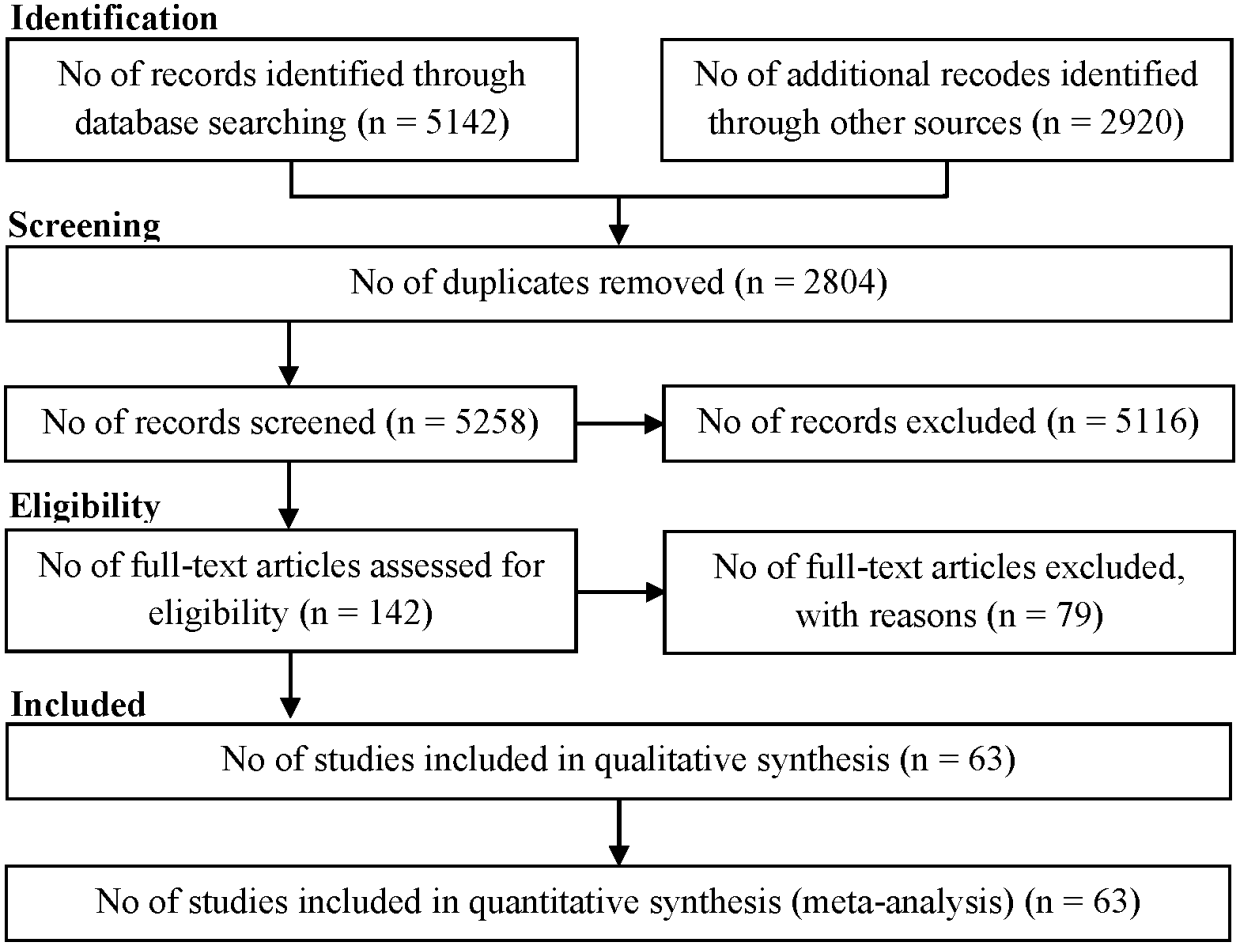
Flow of information through the different phases of the systematic review.

We identified a total of 8062 references, including 5142 articles through searching the electronic databases until May 2015 and 2920 articles through screening the reference list of included studies. We excluded 2804 duplicates using EndNote reference manager and 5116 ineligible studies through reading titles and abstracts. Accordingly, we retrieved 142 references for further assessment. We excluded 79 references because they did not meet the inclusion criteria of this meta-analysis. Finally, 63 references remained for meta-analysis (Fig 1) including 25 cohort studies, 3 case-control studies, and 35 cross-sectional studies involving 8,063,634 participants. Fifty-eight studies published in English, two in Spanish [24–26], and three in Korean [27–29] (Table 1).

**Table 1:** Sumarry of the studies results.

1^st^ author, year	Country	Population	Age mean	Sex	Design	Sample	Quality	Suicidal behaviors
Afifi 2007	Canada	General	12.50	Both	Cross-sectional	2,090	High	Ideation, attempt
Almeida 2012	Australia	General	71.83	Both	Cross-sectional	21,290	Low	Ideation
Angsta 1998	Switzerland	Veteran	19.50	Male	Cohort	2,782	Low	Death
Beratis 1997	Greece	General	33.53	Both	Case-control	200	High	Attempt
Berlin 2015	USA	General	43.04	Both	Cross-sectional	34,653	High	Attempt
Boden 2008	New Zealand	General	19.88	Both	Cohort	1,041	High	Ideation, attempt
Bohnert 2014	USA	Veteran	62.09	Both	Cohort	4,863,086	High	Death
Bolton 2010	USA	General	53.46	Both	Cross-sectional	34,653	High	Attempt
Botega 2010	Brazil	General	49.30	Both	Cross-sectional	4,328	High	Ideation
Bronisch 2008	Switzerland	General	19.00	Both	Cohort	482	High	Ideation, attempt
Clarke 2010	USA	General	32.90	Both	Cohort	1,292	High	Ideation
Dervic 2007	Austria	General	15.40	Both	Cohort	214	Low	Ideation
Deveci 2005	Turkey	General	38.20	Both	Cross-sectional	1,086	High	Ideation, attempt
Donald 2006	Australia	General	21.00	Both	Case-control	475	Low	Attempt
Eaton 2011	USA	General	16.50	Female	Cohort	6,322	Low	Ideation, attempt
Epstein 2009	USA	General	15.00	Both	Cross-sectional	13,917	Low	Ideation, plan, attempt
Garrison 1993	USA	General	16.00	Both	Cross-sectional	3,764	High	Ideation, plan, attempt
Goodwin 2013	Japan	Veteran	31.31	Both	Cross-sectional	6,514	High	Ideation
Hallfors 2004	USA	General	16.30	Both	Cross-sectional	18,924	High	Ideation
Han 2009	Korea	General	16.00	Both	Cross-sectional	70,486	High	Ideation, attempt
Hemenway 1993	USA	General	47.17	Female	Cohort	121,700	Low	Death
Hemmingsson 2003	Sweden	Veteran	19.00	Male	Cohort	49,323	High	Death
Hintikka 2009	Finland	General	45.60	Both	Cohort	1,339	Low	Ideation
Hockenberry 2010	USA	General	16.39	Both	Cross-sectional	575	Low	Ideation
Husky 2013	France	General	46.86	Both	Cross-sectional	27,653	Low	Ideation, attempt
Iwasaki 2005	Japan	General	52.23	Male	Cohort	45,209	High	Death
Jee 2011	Korea	General	46.56	Both	Cohort	1,234,927	High	Death
Juan 2010	China	General	15.73	Both	Cross-sectional	4,644	Low	Ideation, attempt
Kessler 2007	USA	General	≥18	Both	Cross-sectional	5,692	High	Ideation, plan, attempt
Kessler 2009	USA	General	≥15	Both	Cohort	5,001	High	Ideation, plan, attempt
Kim 2013	Korea	General	44.91	Both	Cross-sectional	17,065	High	Ideation, attempt
Kokkevi 2012	Europe	General	16.00	Both	Cross-sectional	45,086	High	Attempt
Kumar 2012	Canada	General	40.24	Male	Cross-sectional	6,694	Low	Ideation
Lee 2014	Korea	General	54.49	Both	Cross-sectional	2,349	High	Ideation
Leistikow 2000	USA	General	46.00	Both	Cohort	82,461	High	Attempt, death
Legleye 2010	France	General	24.66	Both	Cross-sectional	4,075	High	Ideation
Lucas 2013	USA	General	41.65	Both	Cohort	253,033	High	Attempt, death
McGee 2005	New Zealand	General	19.50	Both	Cross-sectional	764	High	Ideation
Miller 2000a	USA	Veteran	28.00	Male	Cohort	300,000	High	Attempt, death
Miller 2000b	USA	General	57.50	Male	Cohort	51,529	High	Attempt, death
Miller 2011	Mexico	General	14.50	Both	Cross-sectional	3,005	High	Ideation, plan, attempt
Park 2006	Korea	General	15.50	Both	Cross-sectional	1,312	Low	Ideation
Park 2008	Korea	General	16.00	Both	Cohort	71,404	Low	Attempt
Pérez-Amezcua 2010	México	General	16.50	Both	Cross-sectional	12,424	Low	Ideation, attempt
Paffenbarger 1994	USA	General	≥35	Male	Cohort	21,582	High	Attempt, death
Pfaff 2005	Australia	General	72.20	Both	Cross-sectional	1,061	High	Ideation
Riala 2009	Finland	General	≥14	Both	Cohort	7,995	High	Attempt
Rudatsikira 2007	Uganda	General	14.93	Both	Cross-sectional	1,970	Low	Ideation
Rudatsikirb 2007	Zimbabwe	General	14.64	Both	Cohort	1,506	Low	Ideation
Schneider 2005	Germany	General	51.08	Both	Case-control	552	High	Attempt, death
Schneider 2014	Germany	General	48.40	Both	Cohort	12,888	High	Attempt, death
Silva 2015	Brazil	General	15.50	Both	Cross-sectional	2,207	High	Ideation, plan, attempt
Smith 1992	USA	General	35–57	Male	Cohort	361,662	High	Attempt, death
Sonderman 2015	USA	General	40–79	Both	Cohort	73,422	High	Attempt, death
Tanskanen 2000	Finland	General	43.40	Both	Cohort	36,527	High	Attempt, death
Ursoniu 2009	Romania	General	16.79	Both	Cross-sectional	2,908	Low	Attempt
Valdivia 2015	Chile	General	15.90	Both	Cross-sectional	195	Low	Attempt
Wilson 2012	Seychelles	General	14.00	Both	Cross-sectional	1,427	Low	Ideation, plan
Wong 2013	USA	General	15.00	Both	Cross-sectional	73,183	High	Ideation, plan, attempt
Woods 1997	USA	General	16.00	Both	Cross-sectional	3,054	High	Attempt
Wu 2004	USA	General	12.90	Both	Cross-sectional	1,458	High	Ideation, attempt
Yi 2011	Korea	General	15.46	Both	Cross-sectional	17,783	Low	Attempt
Zhang 2005	USA	General	28.92	Both	Cross-sectional	7,391	High	Attempt

Thirty-five studies addressed the association between smoking and suicidal ideation [25, 28, 30–62], 8 studies addressed the association between smoking and suicide plan [39, 40, 48, 49, 55, 59–61], 30 studies addressed the association between smoking and suicide attempt [25–27, 29, 30, 32, 34, 37–40, 43, 46–50, 55, 59, 61–71], and 16 studies addressed the association between smoking and suicide death [72–86]. The number of studies presented in the forest plots may be more than the total number of included studies. The reason is that some studies reported the association between smoking and different types of suicidal behaviors simultaneously.

### Association between exposure and outcome

**Fig 2:**
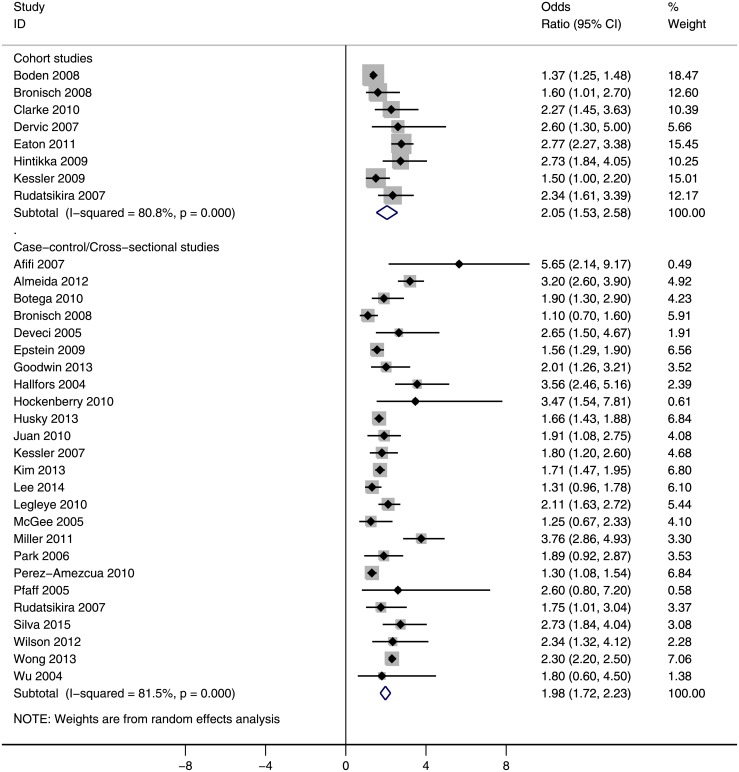
The risk of suicide ideation among current smokers versus nonsmokers. Squares and the horizontal lines represent the measures of effect, e.g. odds ratio or relative risk, and associated confidence intervals for each of the studies and the diamond indicates the summary measure.

The risk of suicidal ideation among current smokers versus nonsmokers is shown in Fig 2. According to this forest plot, there was a significant association between cigarette smoking and suicidal ideation. Current smoking was reliably associated with suicidal ideation. The overall estimate of OR was 2.05 (95% CI: 1.53, 2.58) based on cohort studies and 1.98 (95% CI: 1.72, 2.23) based on case-control/cross-sectional studies. The between-study heterogeneity was high for both groups of studies (P<0.001, I^2^ = 80.8% and I^2^ = 81.5%, respectively).

We also assessed the risk of suicidal ideation among former smokers versus nonsmokers (no figure is given). The former smokers were at higher risk for suicidal ideation. The overall estimate of OR was 1.65 (95% CI: 1.09, 2.22; 2 studies; I^2^ = 0.0%; P = 0.875) on the basis of cohort studies and 1.26 (95% CI: 1.06, 1.46; 5 studies; I^2^ = 69.1%.1%; P = 0.012) according to case-control/cross-sectional studies.

**Fig 3:**
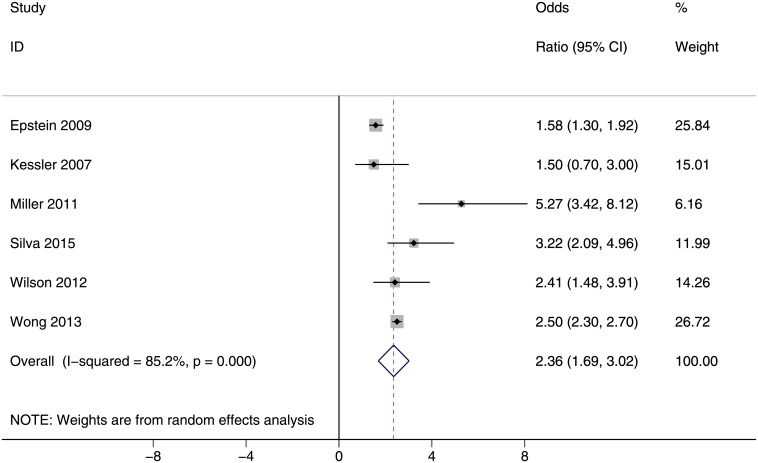
The risk of suicide plan among current smokers versus nonsmokers. Squares and the horizontal lines represent the measures of effect, e.g. odds ratio or relative risk, and associated confidence intervals for each of the studies and the diamond indicates the summary measure.

The risk of suicide plan among current smokers versus nonsmokers is given in Fig 3. This figure indicates a significant association between smoking and suicide plan. Based on this forest plot, the overall estimate of OR was 2.36 (95% CI: 1.69, 3.02). The between-study heterogeneity was high (I^2^ = 85.2%; P<0.001). No study reported the risk of suicide plan among former smokers.

**Fig 4:**
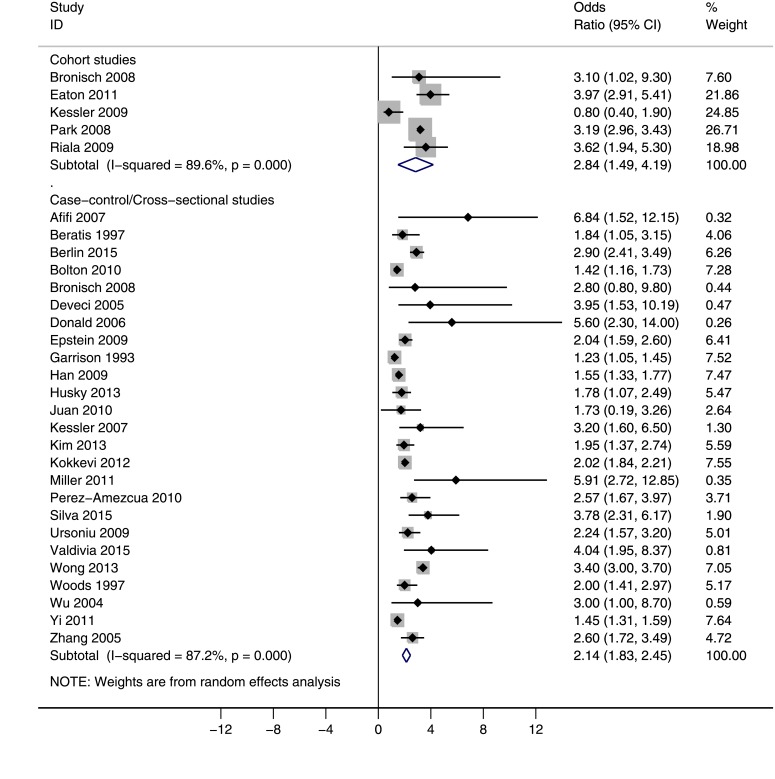
The risk of suicide attempt among current smokers versus nonsmokers. Squares and the horizontal lines represent the measures of effect, e.g. odds ratio or relative risk, and associated confidence intervals for each of the studies and the diamond indicates the summary measure.

The risk of suicide attempt among current smokers versus nonsmokers is shown in Fig 4. According to this forest plot, compared to nonsmokers, the current smokers were at higher risk of suicide attempt. The overall estimate of OR was 2.84 (95% CI: 1.49, 4.19) based on cohort studies and 2.14 (95% CI: 1.83, 2.45) based on case-control/cross-sectional studies. The between-study heterogeneity was high for both groups of studies (P<0.001, I^2^ = 89.6%, I^2^ = 87.2%, and 35.0%, respectively).

We also explored the risk of suicide attempt among former smokers versus nonsmokers (no figure is given). The association was not statistically significant. The overall estimate of OR was 1.40 (95% CI: 0.50, 3.30; 2 studies; I^2^ = 46.6%; P = 0.171) based on cohort studies and 1.09 (95% CI: 0.75, 1.43; 5 studies; I^2^ = 54.8%; P = 0.065) based on case-control/cross-sectional studies.

**Fig 5:**
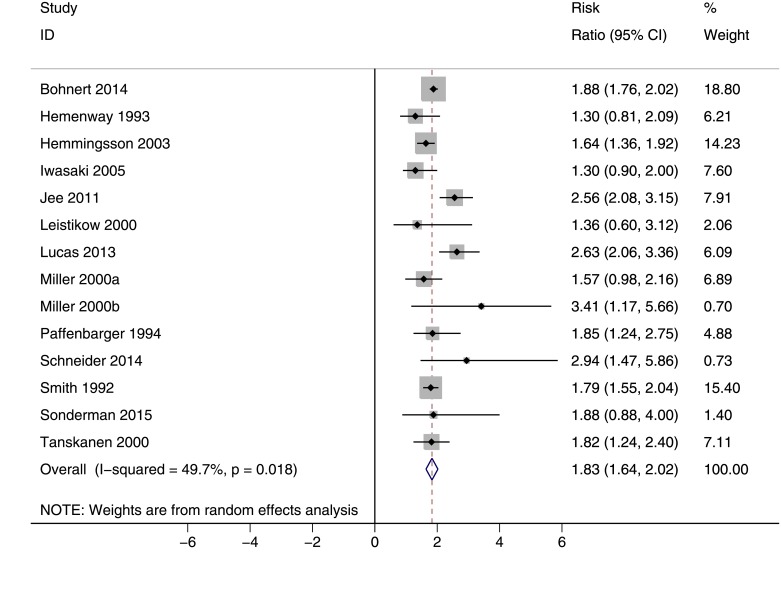
The risk of suicide death among current smokers versus nonsmokers. Squares and the horizontal lines represent the measures of effect, e.g. odds ratio or relative risk, and associated confidence intervals for each of the studies and the diamond indicates the summary measure.

The risk of suicide death among current smokers versus nonsmokers is given in Fig 5. According to this forest plot, the overall estimate of RR was 1.83 (95% CI: 1.64, 2.02). The between-study heterogeneity was moderate (I^2^ = 49.7%; P = 0.018).

We investigated the risk of suicide death in former smokers versus nonsmokers (no figure is given). The association was not statistically significant (RR = 1.33; 95% CI: 0.89, 1.78; 7 studies; I^2^ = 77.6%; P<0.001).

### Publication bias

Publication bias was assessed using Begg's and Egger's tests. On the basis of these statistical tests, there was no evidence of publication bias among studies addressing the association between smoking and suicidal ideation (P = 0.306 and P = 0.200), suicide plan (P = 0.621 and P = 0.823), suicide attempt (P = 0.205 and P = 0.821), and suicide death (P = 0.322 and P = 0.484), respectively.

**Fig 6:**
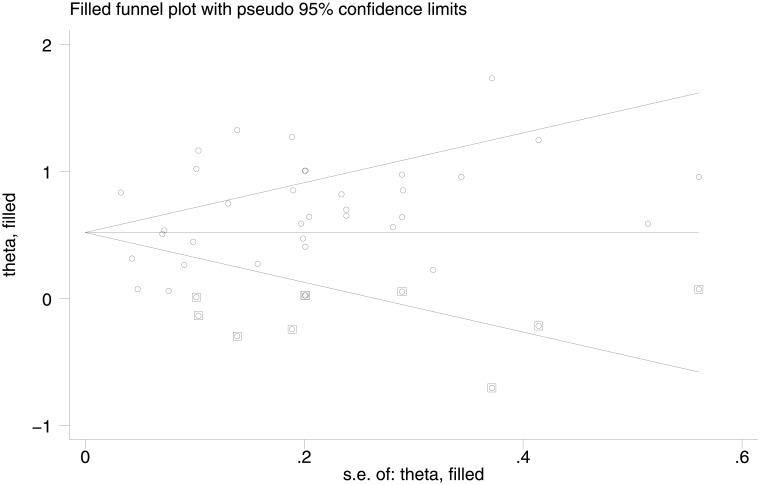
Trim and Fill analysis estimating the number possible missing studies for the association between smoking and suicide ideation. The squares represent the possible missing studies.

We also explored the possibility of publication bias using Trim and Fill method (Fig 6). This statistical method accounts for publication bias in meta-analysis. The method, a rank-based data augmentation technique, formalizes the use of funnel plots, estimates the number and outcomes of missing studies, and corrects the meta-analysis to incorporate the theoretical missing studies [21]. Based on Trim & Fill method, there was evidence of publication bias among the studies addressing the association between suicidal ideation and smoking. On the basis of this method, the uncorrected OR (before adding the possible missing studies) was 2.00; 95% CI: 1.78, 2.22) and the corrected OR (after adding 9 possible missing studies) was 1.68 (95% CI: 1.47, 1.92). Although there was evidence of publication bias, however, its effect was not significant.

### Subgroup analysis

The quality of the studies was explored using NOS scale. According to this scale, there were 42 high-quality studies and 21 low-quality ones (Table 1). We performed subgroup analysis based on the quality of studies and compared the results of high-quality studies with low-quality ones (Table 2). There was no significant difference between the two groups.

**Table 2:** The association between current smoking on suicidal behaviors by the quality of the studies.

Suicidal behaviors	n	I^2^	OR (95% CI)
Suicide ideation			
High-quality studies	19	87.4%	1.96 (1.65, 2.27)
Low-quality studies	13	78.6%	2.09 (1.73, 2.45)
Suicide plan			
High-quality studies	5	65.5%	2.47 (1.70, 3.23)
Low-quality studies	2	40.6%	1.78 (1.09, 2.47)
Suicide attempt			
High-quality studies	19	89.5%	2.19 (1.78, 2.60)
Low-quality studies	10	94.7%	2.46 (1.71, 3.21)
Suicide death			
High-quality studies	14	45.0%	1.87 (1.68,2.06)
Low-quality studies	1	-	1.30 (0.66, 1.94)

### Sensitivity analysis

Based on the sequential algorithm, the number of studies that had to be omitted from the meta-analysis to drop I^2^ below the intended threshold (50%) was two for cohort studies addressing the association between smoking and suicide ideation (I^2^ = 10.5%; OR 2.41; 95% CI: 2.02, 2.80) and four for case-control/cross-sectional ones (I^2^ = 41.9%; OR 1.653; 95% CI: 1.48, 1.83). Two studies were omitted to drop I^2^ below the intended threshold for case-control/cross-sectional studies addressing the association between smoking and suicide plan (I^2^ = 22.3%; OR 2.43; 95% CI: 1.99, 2.86). Finally, the number of studies, which were omitted from the meta-analysis to drop I^2^ below the desired threshold, was one for cohort studies addressing the association between smoking and suicide attempt (I^2^ = 0.0%; OR 3.22; 95% CI: 2.99, 3.45) and five for case-control/cross-sectional ones (I^2^ = 38.9%; OR 2.048; 95% CI: 1.80, 2.29).

## Discussion

In this systematic review, we summarized the available evidence from epidemiological studies exploring the association between smoking habits and suicidal behaviors. Our results suggest that both current and former smokers are at higher risk of suicidal ideation, suicide plan, suicide attempt, and suicide death.

Although the results of this meta-analysis confirmed the association between smoking and suicide, it does not mean causation. In other words, suicidal behaviors are more common among current smokers and the prevalence of smoking habits is higher among suicidal individuals. That means smoking is associated with suicide, but it does not necessarily mean smoking causes suicide. It is still unclear whether smoking influences suicidal behaviors through a biological pathway of smoking itself or whether there is collinearity between smoking and other covariates that are associated with suicide such as psychosocial risk factors or high risk behaviors [9, 11]. Previous epidemiological studies indicated that smoking is part of a pattern of problematic behavior that is linked to various psychopathological disturbances. Several studies reported that smoking is generally associated with psychological disorders and high risk behaviors such as substance and alcohol abuse, sexual and physical abuse, which are considered as major causes of suicide [7, 9, 11, 82, 87]. In addition, a meta-analysis which was conducted by Sankaranarayanan et al in 2015 reported that smoking was significantly associated with an increased risk of suicidality among individuals with a severe mental illness. According to this meta-analysis, the OR of suicidality among psychosis estimated to be 2.12 (95% CI 1.67, 2.7). [88].

Darvishi et al [9] conducted a meta-analysis in 2015 to estimate the alcohol-related risk of suicide. They assessed 31 epidemiological studies and reported that alcohol use dependence increases the risk of suicidal ideation 1.86 fold (95% CI: 1.38, 2.35), the risk of suicide attempt 3.13 fold (95% CI: 2.45, 3.81); and the risk of suicide death 2.59 fold (95% CI: 1.95, 3.23). Another meta-analysis was conducted by Poorolajal et al [11] in 2016 to address the association between substance use disorder and suicidal behaviors. They assessed 43 epidemiological studies and concluded that the substance use disorder was significantly associated with an increased risk of suicidal ideation (OR 2.04; 95% CI: 1.59, 2.50); suicide attempt (OR 2.49; 95% CI: 2.00, 2.98) and suicide death (OR 1.49; 95% CI: 0.97, 2.00). It is likely that part of the association between smoking and suicide reported in this meta-analysis may be explained by the confounding effects of these well-known risk factors. However, another part of the association may be the result of the effect of smoking itself that may increase the risk of suicide through a biological pathway. It is suggested that smoking can significantly decrease the activity of the serotonergic system of the human hippocampus and may reduce brain serotonin function which is negatively related to risk of suicide [89, 90]. Furthermore, nicotine is a potent activator of the hypothalamus, pituitary, adrenal (HPA) axis and is able to activate the attenuated responsiveness of the HPA axis to psychological stress. On the other hand, hyperactivity of the HPA axis is supposed to be a risk factor for suicidal behaviors [91, 92].

This systematic review had a few limitations. First, the results of this meta-analysis are based on the data extracted from observational studies which are associated with the inherent biases that cannot be changed. Furthermore, we were unable to confirm the causal effect of smoking on suicide. Despite these limitations, this meta-analysis could efficiently estimate the relationship between smoking habits and suicidal behaviors. We provided a wide search strategy to enhance the sensitivity of the search to encompass as many studies as possible. We considered all types of observational studies irrespective of age, country, race, publication date, and language. We assessed 8060 articles and included 63 studies with 8,063,634 participants. Therefore, the evidence was sufficient to make a reliable conclusion regarding the objective of this review for estimating the association between smoking habits and suicidal behaviors.

We have high confidence based on the results of studies included in this meta-analysis that smoking is significantly associated with suicidal behaviors. Accordingly, further research is very unlikely to have an important impact on our confidence about the association and is unlikely to change the overall effect. However, limited number of studies explored the dose-response relationship between smoking and suicide. Therefore, further evidence is required to assess the association between the number of cigarettes/day and suicide outcomes.

Our results indicated a smoking-suicide connection. Although this association does not imply causation, but smoking may increase the risk of suicide through affecting on the biological pathways that may increase the risk of suicide or through relationship with other high-risk behaviors such as alcohol and drug dependence. Accordingly, our findings suggest that smoking should be considered as a contributing factor for suicide and smoking prevention and cessation should also be the target of suicide prevention programs.

## Conclusion

There is sufficient evidence based on the current epidemiological studies that smoking is significantly associated with an increased risk of suicidal behaviors. Therefore, smoking can be considered as a contributing factor for suicide, although this association does not necessarily imply causation.

## Supporting Information

S1 PRISMA Checklist (DOC)

S1 Excluded Studies With Reasons (DOC)
