# Supplementary material for: Smoking and Suicide: A Meta-Analysis
Source: PLoS One. 2016 Jul 8;11(7):e0156348. doi: 10.1371/journal.pone.0156348 (PMC4938402; doi:10.1371/journal.pone.0156348)
Supplement: S1 Excluded Studies With Reasons — (DOC) [file pone.0156348.s002.doc]

**Appendix 2**: Characteristics of the excluded studies

| **Row** | **Study** | **Reason for exclusion** |
| --- | --- | --- |
|  | Barbosa 2014 | There has been no distinction between the types of suicide. |
|  | Young 2014 | A review article. |
|  | Lejoyeux 2013 | Aggressive behavior rather than suicide behavior. |
|  | Hooman 2013 | Duplicate publication. |
|  | Li 2012 | A meta-analysis. |
|  | Grucza 2015 | The association between smoking and suicide was not investigated. |
|  | Balbuena 2014 | High smokers were compared with low smokers rather than no smokers. |
|  | Swahn 2012 | The age of initiation rather than smoking itself on suicide was investigated. |
|  | Scherrer 2012 | The effect of smoking among offspring on parents' suicide was investigated. |
|  | Li 2012 | A meta-analysis that investigated suicidal behaviors in mainland China. |
|  | Khan 2012 | A non-reliable study with clear wrong OR estimate [OR 7.10; 95% CI: 3.94, 2.83]. |
|  | Gilreath 2012 | The effect of other covariates on smoking and suicidality were investigated. |
|  | Covey 2012 | Types of suicide behaviors were not reported separately. |
|  | Yaworski 2011 | Effect of nicotine dependence rather than smoking on suicide was investigated. |
|  | Toprak 2011 | The effect of smoking on self-harm rather than suicide was investigated. |
|  | Nenadic-Sviglin 2011 | The association between smoking and suicide was not explored. |
|  | Kim 2011 | A letter to the editor. |
|  | Berlin 2011 | The association between suicide and duration of smoking abstinence rather than smoking was explored. |
|  | Godber 1970 | The effect of smoking was assessed on self-inflicted injuries. |
|  | Gollust 2008 | The effect of smoking on self-injury without intending to kill oneself was investigated. |
|  | Altamura 2003 | The association of variables other than smoking and suicide was investigated. |
|  | Andreasson 1991 | The association between high-risk behavior and mortality rather than suicide was investigated. |
|  | Aubin 2011 | A letter to the editor. |
|  | Baethge 2009 | Suicide ideation and attempts were not reported separately. |
|  | Breslau 2005 | Suicide ideation and attempts were not reported separately. |
|  | Cerel 2005 | Suicidal behavior in the family and adolescent risk behavior was evaluated. |
|  | Clayton 1998 | Editorial |
|  | Espinoza 2010 | Suicide ideation and attempts were not reported separately. |
|  | Fishbain 2009 | The effect of heavy coffee consumption and alcohol use on suicide was investigated. |
|  | Fokkens 2007 | Editorial |
|  | Freitas 2008 | Suicide behavior was investigated among pregnant teenagers versus non-pregnant teenagers |
|  | Gilreath 2009 | Suicide ideation was considered as a predictor of lifetime smoking. |
|  | Koivumaa-Honkanen 2001 | The combined effect of smoking and alcohol on suicide behavior was investigated. |
|  | Lester 1998 | A letter to the editor. |
|  | Lineberry 2009 | The association between smoking and suicide was not reported. |
|  | Menti 2007 | This study was eligible, but was excluded because of unreliable results (lower limits of CIs were higher than point estimates). |
|  | Millares 2010 | The association between smoking and suicide was not reported. |
|  | Patten 2003 | The association between smoking and suicide was not reported. |
|  | Petronis 1990 | The association between smoking and suicide was not reported. |
|  | Riala 2009 | The effect of nicotine dependence rather than smoking on suicide was investigated. |
|  | Rihmer 2007 | The association between smoking and suicide was not reported. |
|  | Shaffer 1993 | A letter to the editor. |
|  | Shaffer 1993 | Editorial. |
|  | Shah 2008 | The association between smoking and suicide was not reported. |
|  | Shah 2010 | The association between smoking and suicide was not reported. |
|  | Sheikh 2000 | A letter to the editor. |
|  | Tanskanen 2000 | Double publication. |
|  | Wilcox 2004 | The effect of early start smoking on suicide behavior was investigated. |
|  | Zajicek 1993 | Editorial. |
|  | Boudreaux 2005 | The association between smoking and suicide was not reported. |
|  | Choquet 1990 | The association between smoking and suicide was not reported. |
|  | Smith 1992 | Duplicated. |
|  | Patten 2003 | Duplicated. |
|  | Innamorati 2011 | The association between smoking and suicide was not reported. |
|  | Bhopal 1992 | A letter to the editor. |
|  | Franca 2015 | This paper was not related to suicide behavior. |
|  | Tempier 2015 | Non-smokers were not separated from irregular smokers. |
|  | Schneider 2011 | Double publication. |
|  | Rebholz 2011 | It assessed ever smokers rather than current and former smokers in separate. |
|  | Tverdal 1993 | The association between smoking and suicide death was assessed for every 10 cigarette smoking. |
|  | He 2014 | Suicide ideation and attempts were not reported separately. |
|  | Bourgkard 2008 | The association between smoking and suicide was not reported. |
|  | Barbosa 2014 | Suicide ideation and attempts were not reported separately. |
|  | Epstein 2010 | Duplicated. |
|  | Baek 2013a | Assessed the association between suicide and smoking among patients with mental disorder. |
|  | Castro-Diaz 2013 | Assessed the association between suicide and smoking among patients with mental disorder. |
|  | Ducasse 2015 | Assessed the association between suicide and smoking among patients with mental disorder. |
|  | Keizer 2009 | Assessed the association between suicide and smoking among patients with mental disorder. |
|  | Makikyro 2004 | Assessed the association between suicide and smoking among patients with mental disorder. |
|  | Malone 2003 | Assessed the association between suicide and smoking among patients with mental disorder. |
|  | Oquendo 2004 | Assessed the association between suicide and smoking among patients with mental disorder. |
|  | Ostacher 2009 | Assessed the association between suicide and smoking among patients with mental disorder. |
|  | Reutfors 2009 | Assessed the association between suicide and smoking among patients with mental disorder. |
|  | Riala 2007 | Assessed the association between suicide and smoking among patients with mental disorder. |
|  | Sankaranarayanan 2014 | Assessed the association between suicide and smoking among patients with mental disorder. |
|  | Schneider 2009 | Assessed the association between suicide and smoking among patients with mental disorder. |
|  | Sharifi 2013 | Assessed the association between suicide and smoking among patients with mental disorder. |
|  | Tanskanen 1998 | Assessed the association between suicide and smoking among patients with mental disorder. |
|  | Zhang 2013 | Assessed the association between suicide and smoking among patients with mental disorder. |
